# HydroMg attenuates doxorubicin-induced myocardial injury via promoting mitochondrial function

**DOI:** 10.4103/mgr.MEDGASRES-D-25-00179

**Published:** 2026-09-12

**Authors:** Lu Zhang, Yawei Jin, Yuning Zhang, Mohan Li, Xiong Gao, Bin Ma, Xiaonan Ning, Aijun Sun, Wenjiang Ding, Zhiguang Ding, Xiurui Ma, Xiaolei Sun, Jian An

**Affiliations:** 1 The Shanxi Medical University, Taiyuan, Shanxi Province, China; 2 Department of Cardiology, Zhongshan Hospital, Fudan University, Shanghai Institute of Cardiovascular Diseases, Shanghai, China; 3 State Key Laboratory of Cardiovascular Diseases, Zhongshan Hospital, Fudan University, Shanghai, China; 4 NHC Key Laboratory of Ischemic Heart Diseases, Shanghai, China; 5 Key Laboratory of Viral Heart Diseases, Chinese Academy of Medical Sciences, Shanghai, China; 6 National Clinical Research Center for Interventional Medicine, Shanghai, China; 7 Institutes of Biomedical Sciences, Fudan University, Shanghai, China; 8 Shanghai Jiao Tong University, Shanghai, China; 9 Shanghai HydroMg Technology Co., Ltd., Shanghai, China; 10 Department of Cardiology, Shan Xi Cardiovascular Hospital, Taiyuan, Shanxi Province, China; 11 Shanxi Coal Center Hospital, Taiyuan, Shanxi Province, China

**Keywords:** apoptosis, cardioprotection, cardiotoxicity, doxorubicin, ferroptosis, hydrogen, HydroMg, mitochondrial dysfunction, mitochondrial homeostasis, ROS

## Abstract

In contrast to molecular hydrogen’s intrinsic limitations, HydroMg’s structural optimization shortens gastrointestinal transit, reducing hydrogen dissipation while enhancing cellular bioavailability. However, it remains unclear whether it also provides protection against cardiac injury. Here, we investigated the cardioprotective effect of HydroMg, a sustained-release hydrogen donor, against doxorubicin-induced cardiac injury. *In vitro* studies showed that 1 μg/mL HydroMg treatment attenuated doxorubicin-induced cardiomyocyte death by inhibiting apoptosis, reducing reactive oxygen species production, and restoring mitochondrial membrane potential. The cardioprotective effect of HydroMg (20, 100, 500, and 1000 mg/kg) was further validated in doxorubicin-induced mice. The 100 mg/kg HydroMg group exhibited 35.7% lower mortality than doxorubicin-treated controls. Additionally, HydroMg treatment significantly improved cardiac function and increased left ventricular ejection fraction by 12.44% compared with the doxorubicin-only group. Mechanistically, HydroMg treatment alleviated mitochondrial dysfunction in the doxorubicin-induced cardiotoxicity mouse model by scavenging reactive oxygen species to dually suppress ferroptosis (via solute carrier family 7 member 11/glutathione peroxidase 4 axis upregulation) and apoptosis (characterized by caspase-3 downregulation and Bcl-2 upregulation). Overall, HydroMg treatment inhibits doxorubicin-induced mitochondrial dysfunction by attenuating reactive oxygen species overproduction, thereby suppressing ferroptosis and apoptosis.

## Graphical abstract

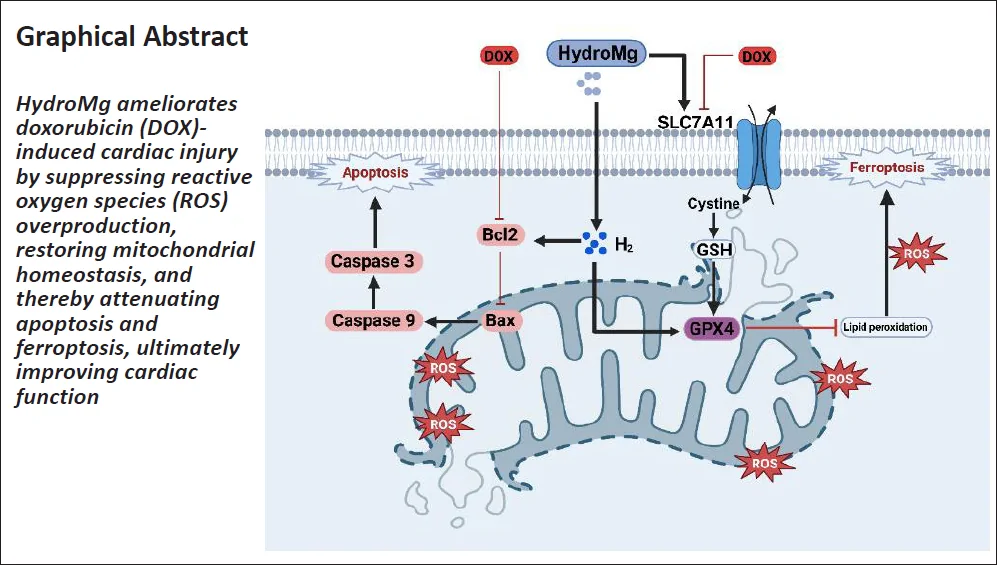

## Introduction

Doxorubicin, a potent anthracycline-class antibiotic, has been clinically used since the 1970s to treat various malignancies.^1^ However, its therapeutic utility is limited by dose-dependent cardiotoxicity, the most common and prognostically significant side effect of anthracycline chemotherapy.^2^,^3^,^4^ Notably, cumulative doxorubicin exposure exceeding 550 mg/m^2^ causes clinically significant cardiotoxicity in approximately 26% of patients, imposing dose-limiting constraints on its therapeutic application in oncology.^5^,^6^,^7^ Elucidating the pathogenesis of doxorubicin-induced cardiotoxicity is critical for developing novel cardioprotective strategies, highlighting the need for mechanistic breakthroughs to advance targeted therapies.

Doxorubicin-induced cardiotoxicity involves mitochondrial dysfunction (specifically complex I redox cycling), oxidative stress, and multimodal cell death pathways.^8^,^9^,^10^ The drug disrupts mitochondrial biogenesis and electron transport chain activity, producing pathological levels of superoxide anions and reactive oxygen species (ROS) that impair adenosine triphosphate (ATP) synthesis while oxidatively damaging DNA, proteins, and lipid membranes.^10^,^11^,^12^,^13^ Cardiac antioxidant system dysfunction initiates pathological accumulation of hydrogen peroxide/superoxide anion,^14^ driving self-propagating oxidative cascades. These synergize with calcium overload to trigger mitochondrial permeability transition pore-mediated^15^ mitochondrial apoptosis/ferroptosis,^16^ culminating in collapsed antioxidant defenses, irreversible membrane depolarization, and cardiomyocyte death.^10^,^17^ While mitochondrial-targeted therapies show potential for mitigating doxorubicin-induced cardiotoxicity, the absence of standardized regimens hinders their clinical application.^9^,^17^

Molecular hydrogen mediates pleiotropic therapeutic effects via three primary mechanisms: anti-inflammatory action, apoptosis inhibition, and radical scavenging.^18^,^19^ Current preclinical delivery approaches include oral hydrogen-rich water, inhaled hydrogen gas, and intravenous injection of H_2_-saturated saline.^20^ However, the dose-dependent hydrogen therapy necessitates optimized delivery systems to maximize therapeutic outcomes.^21^ HydroMg is a sub-200-nm magnesium compound that enables sustained hydrogen (H_2_) release, reducing gastrointestinal retention and enhancing therapeutic efficacy. In contrast to molecular hydrogen’s intrinsic limitations, HydroMg’s structural optimization shortens gastrointestinal transit, reducing hydrogen dissipation while enhancing cellular bioavailability. Based on the central role of mitochondrial dysfunction in doxorubicin-induced cardiomyopathy, we hypothesized that HydroMg confers cardioprotection by alleviating oxidative stress through mitochondrial protection. This study therefore aimed to evaluate its therapeutic potential and elucidate the underlying mechanisms, with a focus on redox homeostasis and programmed cell death.

## Methods

### Cell culture

H9C2 rat embryonic-derived myoblasts (Cat# ZQ0102, RRID:CVCL_0286) were purchased from Shanghai Zhong Qiao Xin Zhou Biotechnology Co. (Shanghai, China). Cells were maintained in Dulbecco’s modified Eagle medium (DMEM) supplemented with 10% fetal bovine serum and 100 U/mL penicillin and were incubated at 37°C in 5% CO_2_ within a humidified incubator (Thermo Fisher Scientific, Waltham, MA, USA).

### Animals

Male C57BL/6 mice (6-8 weeks old, weighted 22 ± 2 g) were obtained from SiPeiFu (Suzhou) Biotechnology Co., Ltd. (Suzhou, China). Animals were acclimatized for 7 days in specific pathogen-free conditions with controlled environmental parameters: temperature 23 ± 1°C, humidity 50 ± 5%, and 12-hour light/dark cycle (08:00–20:00). All animal experiments were conducted in compliance with the *Guide for the Care and Use of Laboratory Animals* (8^th^ edition, 2011)^22^ and approved by the Animal Ethics Committee of Zhongshan Hospital, Fudan University (approval No. ZSYY20240618; dated June 18, 2024). Mice were randomly assigned into: control (*n* = 8), doxorubicin (*n* = 28), doxorubicin + HydroMg (20 mg/kg) (*n* = 9), doxorubicin + HydroMg (100 mg/kg) (*n* = 28), doxorubicin + HydroMg (500 mg/kg) (*n* = 15) and doxorubicin + HydroMg (1000 mg/kg) groups (*n* = 8).

### HydroMg and doxorubicin treatment

To assess the therapeutic efficacy of HydroMg, mice received intraperitoneal administration of HydroMg (dissolved in normal saline) at a concentration gradient (20, 100, 500, and 1000 mg/kg) 4–5 hours prior to doxorubicin challenge (15 mg/kg, intraperitoneal injection). Survival and clinical status were monitored daily until terminal echocardiography on day 6, followed by euthanasia and cardiac tissue collection. In parallel, H9C2 cardiomyocytes at 70–80% confluency received HydroMg (0.1, 1, 10, 100, and 1000 μg/mL) for 6 hours before 24-hour doxorubicin exposure (1 μM). Apoptosis and viability were quantified using standardized assays.

### Mortality assessment

Survival was monitored daily within 6 days following injection. The number of deceased mice in each group was recorded every 24 hours, and the exact time of each death was documented.

### Echocardiographic analysis

Cardiac function was evaluated in mice on day 6 after doxorubicin (Sigma-Aldrich, St. Louis, MO, USA) administration using the Vevo 2100 high-frequency ultrasound system (VisualSonics Inc., Toronto, Canada). Under isoflurane anesthesia (3% induction, 1% maintenance; RWD Life Science, Shenzhen, China), M-mode echocardiographic images of the left ventricle were acquired and analyzed.

### Histological analysis

Mice were anesthetized with pentobarbital sodium (70 mg/kg, intraperitoneal injection) prior to cardiac tissue collection. The harvested hearts were fixed by immersion in 4% paraformaldehyde at 4°C for 24 hours. Following fixation, tissues underwent graded dehydration and paraffin embedding according to standardized histological protocols. Tissues were sectioned at 5-μm thickness. For dual-parameter analysis, sections were subjected to fluorescent wheat germ agglutinin staining (Servicebio, Wuhan, China) to delineate cardiomyocyte membranes and the terminal deoxynucleotidyl transferase dUTP nick-end labeling (TUNEL; Servicebio) assay to quantify programmed cell death. Histological evaluations were performed using digital image analysis software on a fluorescence microscope (Leica SR5, Wetzlar, Germany).

### Cell counting kit-8 assay

Cell viability was assessed using the Cell Counting Kit-8 (CCK-8, Beyotime Biotechnology, Shanghai, China) according to standardized protocols. Briefly, H9C2 cells in the logarithmic growth phase were harvested by trypsinization, centrifuged (187 × *g*, 5 minutes at room temperature), and resuspended in DMEM supplemented with 10% fetal bovine serum and 1% penicillin/streptomycin to achieve a seeding density of 1 × 10^4^ cells/well. Cell suspensions (100 μL) were aliquoted into 96-well plates, with peripheral wells filled with phosphate-buffered saline (PBS) to minimize edge effects. After a 24-hour incubation (37°C, 5% CO_2_) to allow adhesion, cells were treated with freshly prepared HydroMg solutions (0.1–1000 μg/mL in complete medium) for 6, 12, and 24 hours. Following each treatment interval, CCK-8 reagent (10 μL per well) was added to the existing culture medium (100 μL per well) and incubated for 1 hour under physiological conditions. Optical density at 450 nm was measured hourly over 4 hours using a multimode microplate reader (Thermo Scientific Varioskan LUX, Waltham, MA, USA), with background subtraction using blank controls.

### Live cell imaging

H9C2 cardiomyocytes were cultured in 6-well plates (Servicebio) using complete DMEM supplemented with 10% fetal bovine serum under standard conditions (37°C, 5% CO_2_). To systematically evaluate HydroMg’s concentration-dependent bioactivity, cells were exposed to escalating doses (0.1–1000 μg/mL) under two distinct experimental regimens: continuous 24-hour HydroMg treatment to assess proliferation modulation, or 6-hour HydroMg preconditioning followed by 24-hour combinatorial exposure to 1 μM doxorubicin to simulate therapeutic synergy. Quantification of cell death modalities was conducted by time-lapse imaging using the BioTek Lionheart FX Automated Live Cell Imager (BioTek Instruments, Winooski, VT, USA).

### Terminal deoxynucleotidyl transferase dUTP nick-end labeling assay

Cardiomyocyte apoptosis was assessed using a one-step fluorescent apoptosis detection kit (Annexin V-FITC/PI, Beyotime Biotechnology). Nuclei were counterstained with 4’,6-diamidino-2-phenylindole (Beyotime Biotechnology) to facilitate morphological evaluation. Fluorescent signals were acquired at dual excitation wavelengths using a laser scanning confocal microscope, with z-stack imaging performed to ensure optical sectioning accuracy.

### Reactive oxygen species analysis

Following treatment with 1 μg/mL HydroMg and 1 μM doxorubicin, cells were stained with MitoSOX Red mitochondrial superoxide indicator (Invitrogen, Carlsbad, CA, USA). The MitoSOX reagent (5 mM stock) was diluted at 1:10,000 in a cell culture medium to prepare the working solution. Cells were incubated with this solution at 37°C under 5% CO_2_ for 30 minutes in the dark, followed by three washes with warm PBS. Mitochondrial ROS in cardiomyocytes were then visualized using fluorescence microscopy. Separately, cellular ROS levels were assessed using the 2’,7’-dichlorodihydrofluorescein diacetate (CM-H2DCFDA) detection kit (Beyotime Biotechnology). The CM-H_2_DCFDA probe was diluted 1:1000 (5 μM final concentration) and incubated with cells at 37°C for 30 minutes in the dark. Green fluorescence images were acquired using a fluorescence confocal microscope.

### Mitotracker deep red staining

Mitochondrial networks were visualized using Mitotracker Deep Red FM (Invitrogen) according to standardized protocols. Briefly, the fluorescent probe was reconstituted in dimethyl sulfoxide and diluted in complete growth medium (DMEM) to a 1:10,000 working concentration. H9C2 cells were incubated with the staining solution under physiological conditions (37°C, 5% CO_2_) for 20 minutes to enable mitochondrial localization. Following staining, cells were fixed with ice-cold methanol (–20°C) for 10 minutes at 4°C, followed by three sequential washes (5 minutes/wash) with PBS. High-resolution images were acquired using a laser scanning confocal microscope.

### Mitochondrial membrane potential measurement

Mitochondrial membrane potential was evaluated using a commercial fluorometric tetramethylrhodamine ethyl ester (TMRE) assay kit (Beyotime Biotechnology). TMRE working solution (1 μM) was prepared by 1:1000 dilution of the stock reagent in serum-free DMEM. Cells were rinsed with PBS post-treatment and incubated with TMRE solution (1 mL/well) under light-protected conditions (37°C, 5% CO_2_, 30 minutes). Following staining, cells underwent two washes with pre-warmed complete medium and were placed in fresh medium for immediate live-cell imaging. TMRE fluorescence was captured using a confocal microscope with 550/575 nm excitation/emission filters.

### Adenosine triphosphate detection

The cell culture medium was aspirated, and the cells were gently washed once with ice-cold PBS. All subsequent steps were maintained on ice. Two hundred microliters of ATP lysis reagent (Beyotime Biotechnology) were added per well of a 6-well plate, and the mixture was gently pipetted up and down several times to ensure complete cellular lysis. The lysate was transferred to pre-chilled 1.5 mL microcentrifuge tubes and centrifuged at 12,000 × *g* for 5 minutes at 4°C. The supernatant, which contained the extracted ATP, was carefully collected into fresh tubes while disturbance of the pellet was avoided. Concurrently, ATP standard solutions were prepared in lysis reagent or an assay-specific diluent across an appropriate concentration gradient for standard curve generation. The ATP detection working solution was prepared immediately before use by diluting the ATP detection reagent with the manufacturer-supplied diluent at the specified ratio (typically 1:4) and was protected from light. One hundred microliters of the working solution was aliquoted into wells of an opaque-walled 96-well plate and incubated at room temperature for 3–5 minutes to deplete background ATP. Subsequently, 20 µL of sample supernatant or ATP standard was added to each well and was immediately mixed by pipetting or brief orbital shaking. After a minimum interval of 2 seconds, relative light units were measured using a luminometer (Thermo Scientific Varioskan LUX). All readings were completed within 10–30 seconds post-mixing, with consistent timing intervals being maintained.

### Western blot analysis

Total protein lysates from cardiomyocytes and cardiac tissues were extracted using radioimmunoprecipitation assay buffer (Beyotime Biotechnology) and quantified via bicinchoninic acid assay assay (Beyotime Biotechnology). Proteins were resolved on 10% or 12.5% sodium dodecyl sulfate-polyacrylamide gels and transferred electrophoretically to polyvinylidene difluoridemembranes (0.22 μm, Millipore, Burlington, MA, USA) via a wet transfer system. After blocking with 5% bovine serum albumin/Tris-buffered saline with Tween 20 for 1 hour at room temperature, membranes were incubated overnight at 4°C with the following primary antibodies against Caspase-3 (rabbit, 1:1000, Cell Signaling Technology, Danvers, MA, USA, Cat#9662S, RRID:AB_331439), Bcl-2 (rabbit, 1:1000, Cell Signaling Technology, Cat#15071, RRID:AB_2744528), translocase of the outer mitochondrial membrane 20 (TOM20; rabbit, 1:1000, Cell Signaling Technology, Cat#42406, RRID:AB_2687663), glutathione peroxidase 4 (GPX4; mouse, 1:5000, Proteintech, Cat#67763, RRID:AB_2909469), solute carrier family 7 member 11 (SLC7A11; rabbit, 1:2000, Proteintech, Cat#26864, RRID:AB_2880661), total oxidative phosphorylation (rodent, 1:250, Abcam, Cambridge, MA, USA, Cat# ab110413, RRID:AB_2629281) and horseradish peroxidase-conjugated monoclonal mouse anti-beta actin (1:5000, KANG CHEN, Shanghai, China, Cat# KC-5A08, RRID:AB_2847811). Following Tris-buffered saline with Tween 20 washes, membranes were probed with horseradish peroxidase-conjugated secondary antibodies (anti-mouse, 1:5000, Abmart, Cat# M21001S, RRID:AB_2713950; anti-rabbit, 1:5000, Abmart, Cat#M21002S, RRID:AB_2713951) for 1 hour at 25°C. Protein bands were visualized using an imaging system (Bio-Rad Laboratories, Hercules, CA, USA).

### Statistical analysis

The sample size (*n* = 8 per group) was determined based on common practices in previous similar studies rather than a formal power analysis. The evaluator was blinded to the group allocation during data collection and analysis. Statistical analyses were conducted using GraphPad Prism 9 (GraphPad Software, Boston, MA, USA, www.graphpad.com). Quantitative data are expressed as mean ± standard error of the mean (SEM). Intergroup comparisons were evaluated by one-way analysis of variance, with significant results further analyzed using Tukey’s *post hoc* test for multiple comparisons, while pairwise analyses were performed using unpaired two-tailed Student’s *t*-tests. Survival curves were generated using the Kaplan–Meier method, and group comparisons were performed with the log-rank test. All experiments included a minimum of three independent biological replicates. Statistical significance was defined as *P* < 0.05.

## Results

### Effect of HydroMg on cardiomyocyte viability

To investigate if prolonged HydroMg exposure affects cardiomyocyte growth, live-cell imaging was used to dynamically monitor H9C2 proliferation across graded HydroMg concentrations (0.1, 1, 10, 100, and 1000 μg/mL) over 24 hours (**Figure 1A**). Results showed that 1000 μg/mL HydroMg triggered pronounced cell death within 24 hours. Conversely, lower concentrations (0.1–100 μg/mL) maintained proliferation dynamics comparable to the control (Figure 1B). Proliferation rates at 0.1, 1, and 10 μg/mL HydroMg after 6-, 12-, and 24-hour exposures showed no statistical significance versus controls (**Figure 1C–E**). Additionally, we utilized the CCK-8 assay to evaluate the cellular activity of H9C2 cells treated with different concentrations of HydroMg (10, 1, and 0.1 μg/mL) at various time points. CCK-8 assay demonstrated that HydroMg significantly enhanced H9C2 viability, specifically at 6 hours (**Figure 1F–H**). Collectively, these findings indicate that concentrations of HydroMg at 10, 1, and 0.1 μg/mL significantly enhance cellular proliferative capacity without compromising viability.

**Figure 1:**
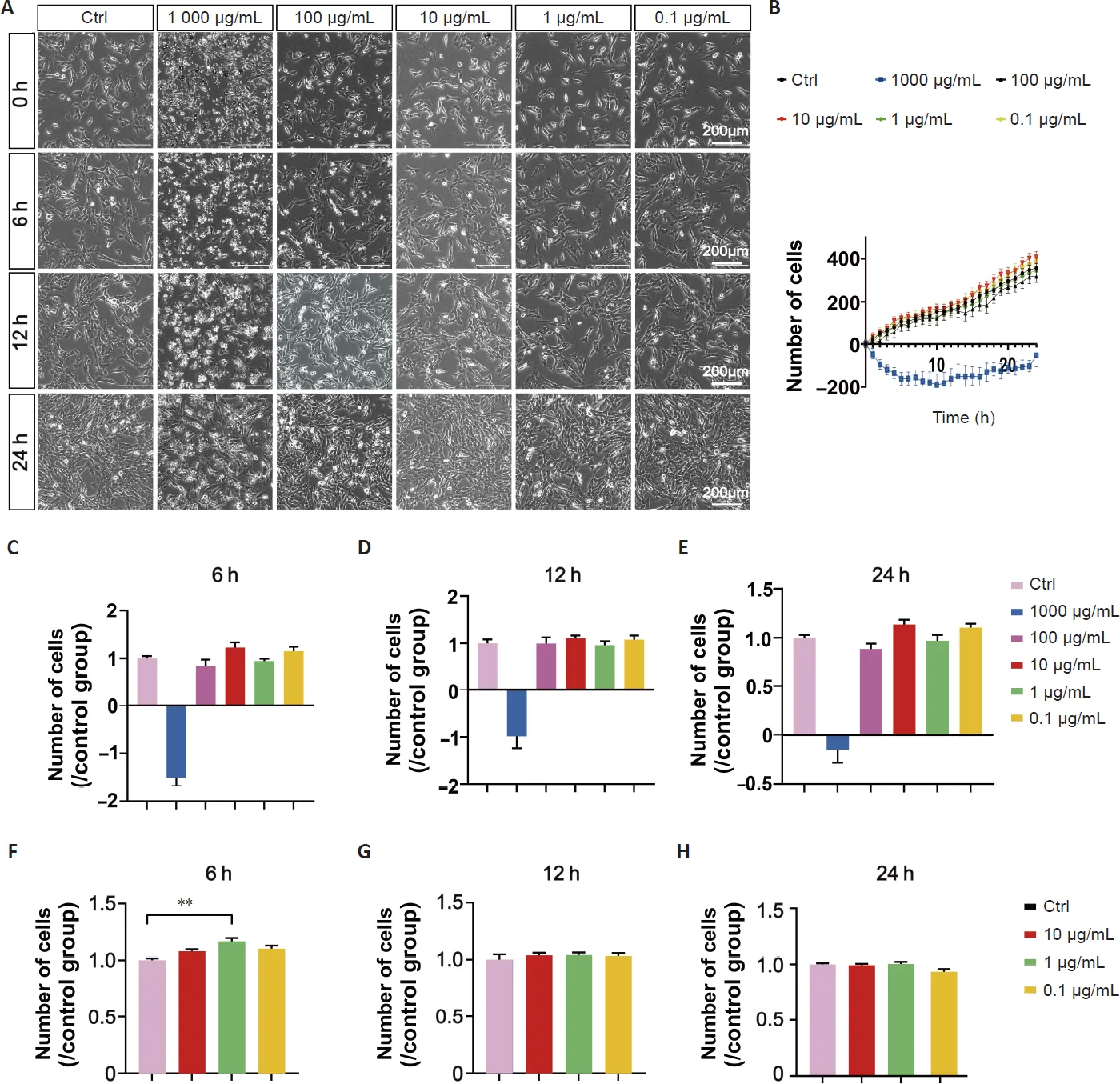
HydroMg improves cardiomyocyte viability. (A) H9C2 cell death was quantified by live-cell imaging following a 24-hour HydroMg treatment (*n* ≥ 3 in each group) Treatment with 1000 μg/mL HydroMg induced significant cell death within 24 hours. In contrast, lower concentrations (0.1–100 μg/mL) showed proliferation rates similar to the control. Scale bars: 200 μm. (B) Effect of HydroMg treatment on H9C2 cells proliferation (24 hours). (C–E) Quantification of H9C2 cells proliferation relative to untreated controls following HydroMg treatment at 6 (C), 12 (D), and 24 hours (E) (*n* ≥ 3 per group). (F–H) H9C2 cells were treated with HydroMg at concentrations of 10, 1, and 0.1 μg/mL for different durations: 6 (F), 12 (G), and 24 hours (H) (*n* ≥ 3 per group). Cell viability was measured using the cell counting kit-8 assay. All results are presented as mean ± SEM. ^**^P < 0.01 (one-way analysis of variance followed by Tukey’s *post hoc* test). Ctrl: Control.

### HydroMg prevents doxorubicin-induced cardiomyocyte death

To assess HydroMg’s cardioprotective effects, H9C2 cells were preconditioned with HydroMg for 6 hours before doxorubicin exposure (**Figure 2A**). In dose-response experiments, cells were preconditioned with graded HydroMg concentrations (10, 1, and 0.1 μg/mL) for 6h followed by a 24-hour challenge with 1 μM doxorubicin. Longitudinal cellular dynamics were quantified via time-lapse live-cell imaging over 24 hours (**Figure 2B**). HydroMg exhibited dose-dependent cardioprotective effects, significantly reducing doxorubicin-induced cell death (**Figure 2C**). HydroMg (1 μg/mL) enhanced proliferation relative to doxorubicin-treated controls (**Figure 2D**). Quantitative TUNEL analysis showed a significant increase in apoptotic indices in doxorubicin-treated cardiomyocytes versus controls. HydroMg co-treatment (1 μg/mL) significantly attenuated doxorubicin-induced apoptosis (**Figure 2E** and **F**). Collectively, these findings indicate that HydroMg treatment mitigates doxorubicin-induced cardiomyocyte death, suggesting its potential protective role against doxorubicin-associated myocardial injury.

**Figure 2:**
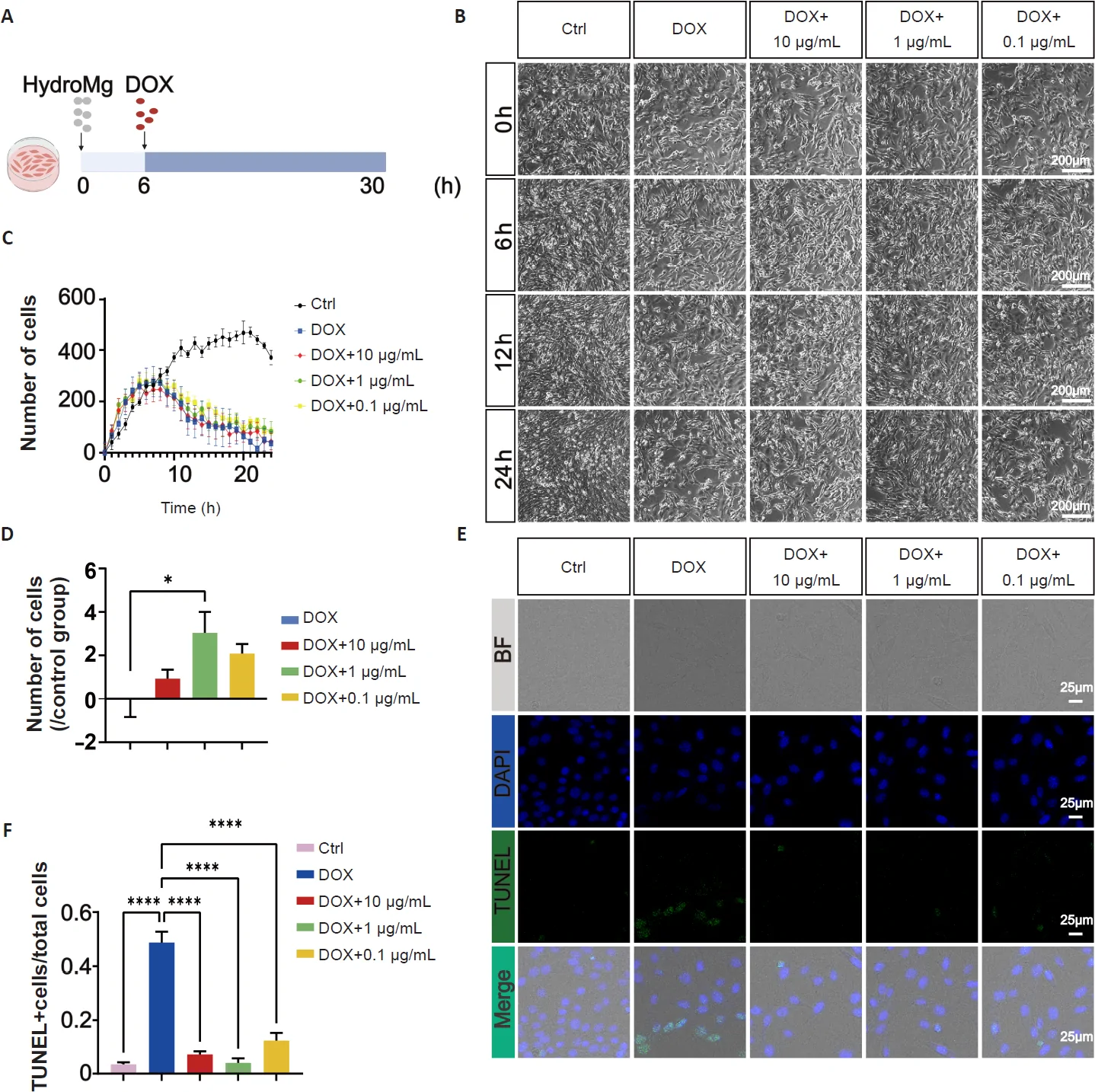
HydroMg inhibits DOX-induced cardiomyocyte death and apoptosis. (A) Experimental scheme. (B) H9C2 cells were treated with HydroMg for 6 hours, followed by doxorubicin intervention for 24 hours, with real-time cell death monitoring via live-cell imaging (*n* ≥ 3 per group). HydroMg resulted in a dose-dependent reduction in doxorubicin-induced cell death, with the 1 μg/mL concentration significantly enhancing cell proliferation compared with the doxorubicin-only group. Scale bars: 200 μm. (C) Dynamic proliferation curve of H9C2 cells during 24-hour DOX intervention following 6-hour HydroMg pretreatment. (D) Relative proliferation rate of H9C2 cells after 6-hour HydroMg pretreatment combined with 24-hour sustained DOX intervention. (E, F) TUNEL staining (green) was performed to detect H9C2 cells apoptosis in each group after HydroMg and DOX treatment (*n* ≥ 3 per group). Scale bars: 25 μm. HydroMg (1μg/mL) significantly reduced doxorubicin-induced apoptosis. All results are presented as mean ± SEM. ^*^P < 0.05, ^****^P < 0.0001 (one-way analysis of variance with Tukey’s *post hoc* test). BF: Bright field; Ctrl: control; DAPI: 4’,6-diamidino-2-phenylindole; DOX: doxorubicin; TUNEL: terminal deoxynucleotidyl transferase dUTP nick-end labeling.

### HydroMg attenuates doxorubicin-induced cardiomyocyte injury by improving mitochondrial function and reducing oxidative stress

To investigate the cytoprotective mechanism of HydroMg (1 μg/mL) against doxorubicin-induced apoptosis, we assessed mitochondrial integrity using three key parameters: structural morphology continuity, membrane potential (ΔΨm) homeostasis, ATP and ROS generation. Analysis of mitochondrial morphology via MitoTracker Deep Red staining revealed significantly increased fragmentation in doxorubicin-treated H9C2 cells compared to controls. HydroMg co-treatment significantly reduced this fragmentation (**Figure 3A** and **B**). Quantitative assessment of ΔΨm showed that doxorubicin-induced severe depolarization relative to vehicle-treated controls. HydroMg co-treatment attenuated this depolarization. (**Figure 3C** and **D**). Furthermore, doxorubicin reduced ATP production in cardiomyocytes, whereas HydroMg co-treatment restored cellular ATP levels (**Additional Figure 1**), suggesting preserved mitochondrial bioenergetic function. Mitochondrial ROS levels, quantified using MitoSOX Red fluorescence, were markedly elevated by doxorubicin compared with controls. HydroMg co-treatment significantly attenuated this increase (**Figure 3E** and **F**). Total cellular ROS, quantified using CM-H2DCFDA fluorescence, was also significantly elevated by doxorubicin versus untreated controls. HydroMg co-administration markedly attenuated this oxidative response compared with doxorubicin alone (**Figure 3G** and **H**). Collectively, these data demonstrate that HydroMg treatment mitigates doxorubicin-induced cardiac injury by preserving mitochondrial function and reducing subsequent cardiac damage.

**Figure 3:**
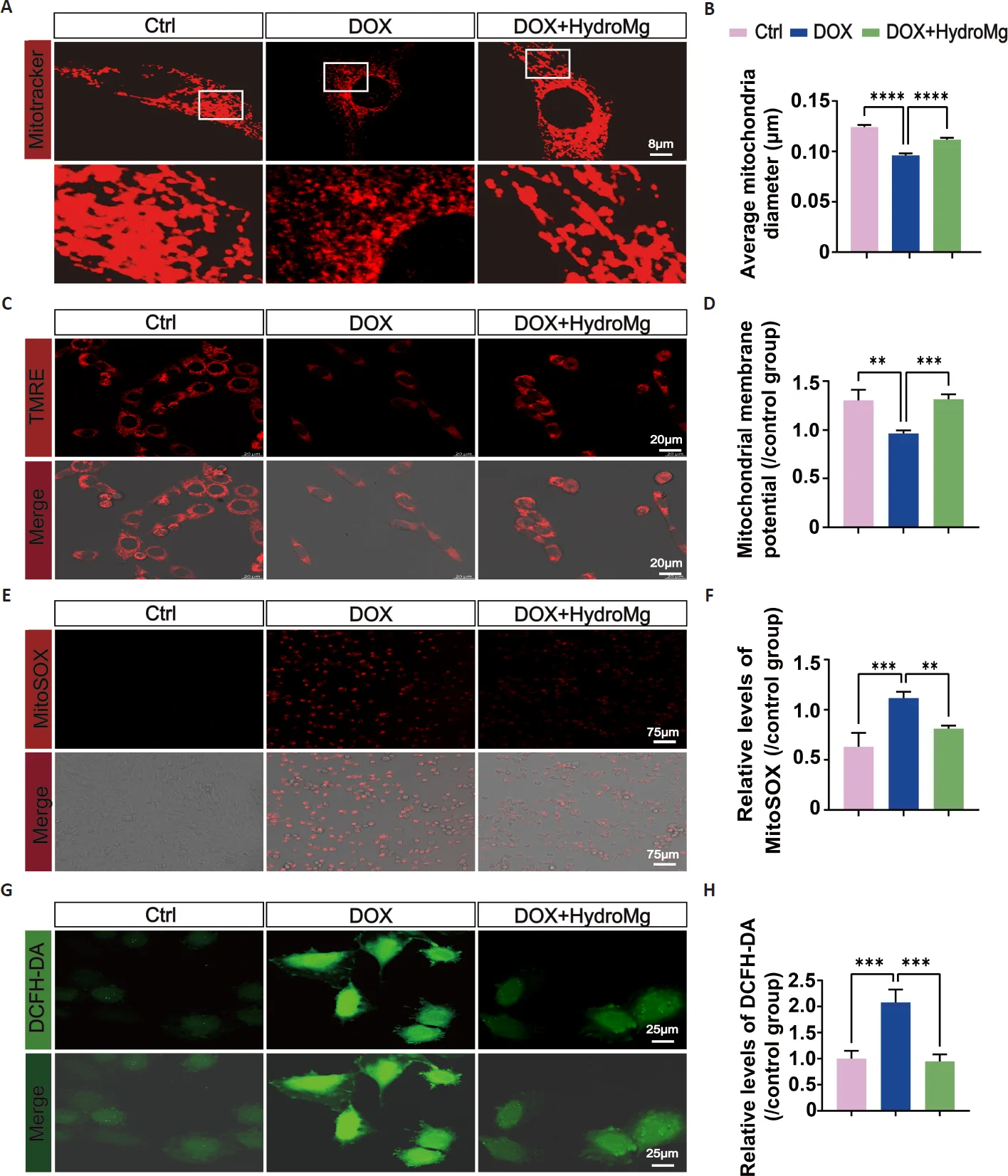
HydroMg alleviates DOX-induced mitochondrial dysfunction and ROS overproduction in cardiomyocytes. (A) Mitochondrial network structures in H9C2 cells were analyzed using MitoTracker Deep Red staining and fluorescence microscopy (*n* ≥ 3 per group). Doxorubicin increased mitochondrial fragmentation in H9C2 cells, which was reduced by HydroMg co-treatment. Scale bar: 8 μm. (B) The average mitochondrial length of mitochondria was analyzed in H9C2 cells from Ctrl, DOX, and DOX + HydroMg groups. (C) Mitochondrial membrane potential in live H9C2 cells was assessed using TMRE staining (*n* ≥ 3 per group). HydroMg attenuated doxorubicin-induced mitochondrial depolarization. Scale bars: 20 μm. (D) TMRE fluorescence intensity was quantified. (E) Mitochondrial ROS production in H9C2 cells was detected using MitoSOX Red staining (*n* ≥ 3 per group). HydroMg reduced doxorubicin-induced mitochondrial ROS elevation. Scale bars: 75 μm. (F) MitoSOX fluorescence intensity was quantified. (G) DCFH-DA staining showing ROS production in H9C2 cells (*n* ≥ 3 per group). HydroMg reduced doxorubicin-induced total ROS elevation. Scale bar: 25 μm. (H) Quantification of DCFH-DA fluorescence intensity. All results are expressed as mean ± SEM. ^**^P < 0.01, ^***^P < 0.001, ^****^P < 0.0001 (one-way analysis of variance followed by Tukey’s *post hoc* test). Ctrl: Control; DCFH-DA: 2’,7’-dichlorodihydrofluorescein diacetate; DOX: doxorubicin; ROS: reactive oxygen species; TMRE: tetramethylrhodamine ethyl ester.

### HydroMg improves cardiac function in the doxorubicin-induced mice

To evaluate the prophylactic cardioprotective efficacy of HydroMg against doxorubicin-induced cardiac injury, mice were pretreated intraperitoneally with different concentrations of HydroMg 4–5 hours prior to doxorubicin administration, establishing an acute chemotherapy-induced cardiomyopathy model. Survival was monitored daily, and cardiac function was assessed on day 6 post-treatment (**Figure 4A**). Doxorubicin increased mortality relative to controls. Paradoxically, higher HydroMg co-administration doses (500 and 1000 mg/kg) further elevated mortality versus doxorubicin monotherapy, whereas 100 mg/kg HydroMg treatment significantly reduced mortality by 35.7% compared with doxorubicin alone (**Figure 4B**). Co-administration of 100 mg/kg HydroMg improved left ventricular systolic function, increasing ejection fraction by 12.44% and fractional shortening by 9.66% relative to doxorubicin monotherapy (**Figure 4C–E** and **Additional Figure 2**). Wheat germ agglutinin staining confirmed doxorubicin-induced cardiomyocyte atrophy, which was attenuated by HydroMg co-administration compared with monotherapy (**Figure 4F** and **G**). These findings demonstrate that 100 mg/kg HydroMg treatment protects against doxorubicin-induced cardiotoxicity by preserving myocardial contractility and improving survival.

**Figure 4:**
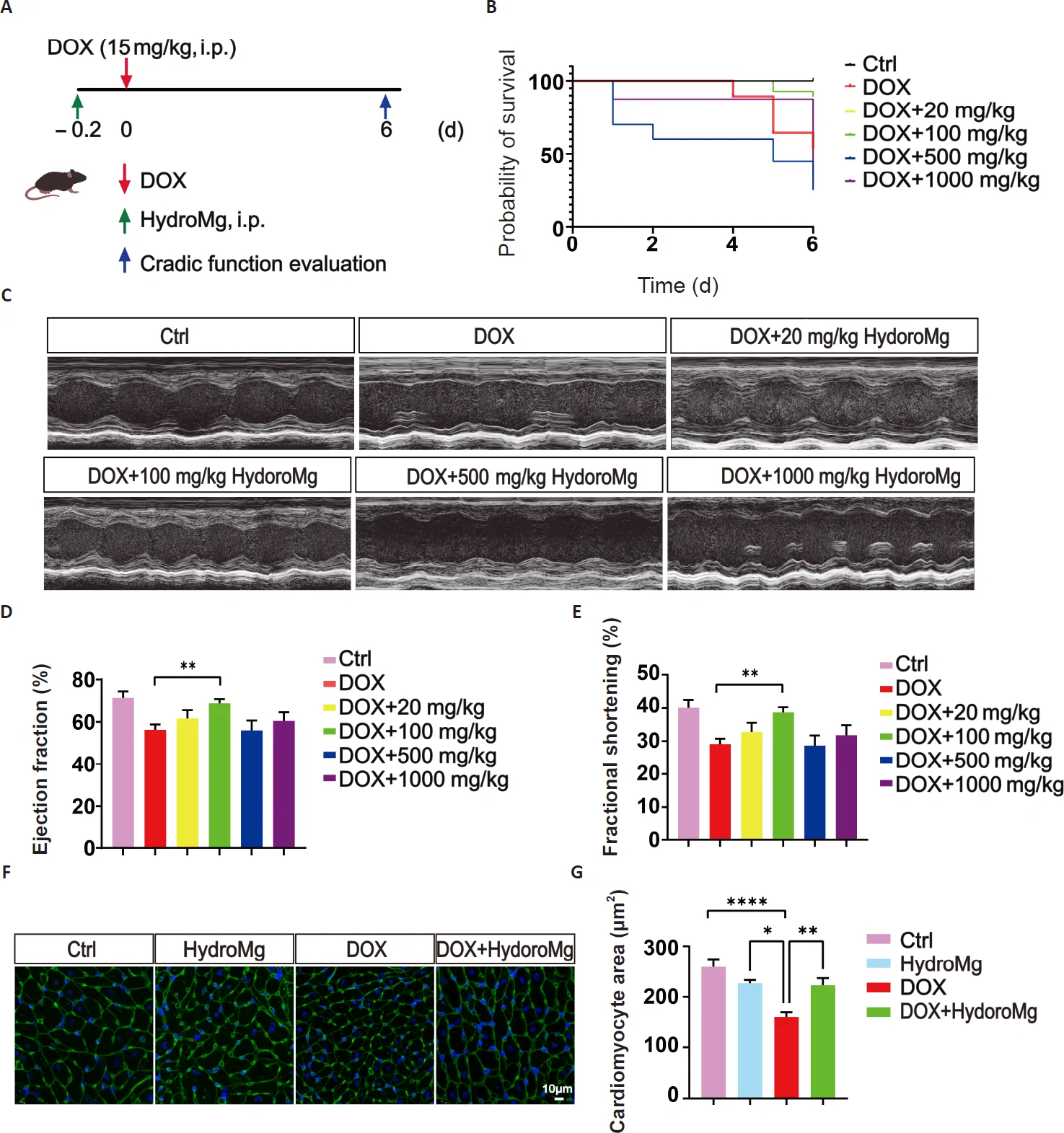
HydroMg ameliorates DOX-induced cardiac contractile dysfunction. (A) Experimental schematic. DOX (15 mg/kg) was administered intravenously on day 0 in 6–8-week-old male C57BL/6 mice, preceded by HydroMg pretreatment via intraperitoneal injection 4–5 hours prior. Cardiac functional parameters were serially monitored until day 6 post-treatment. (B) The survival rate (*n* ≥ 3 per group) using the Kaplan–Meier method. (C) Cardiac function was evaluated by echocardiography. HydroMg (100 mg/kg) improved cardiac function, increasing LVEF by 12.44% and FS by 9.66% *versus* doxorubicin alone. (D) EF. (E) FS. (F) Representative images of wheat germ agglutinin staining in cardiac tissue sections (*n* ≥ 3 per group). HydroMg attenuated doxorubicin-induced cardiomyocyte atrophy (WGA staining). Scale bar: 10 μm. (G) Quantification of cardiomyocyte cross-sectional areas. All results are expressed as mean ± SEM. Statistical analysis was performed using one-way analysis of variance followed by Tukey’s *post hoc* test for multiple comparisons among groups. ^*^P < 0.05, ^**^P < 0.01, ^****^P < 0.0001. Ctrl: Control; DOX: doxorubicin; EF: ejection fraction; FS: fractional shortening; i.p.: intraperitoneal administration; LVEF: left ventricular systolic function; WGA: wheat germ agglutinin.

### HydroMg inhibits apoptosis signaling in the heart of doxorubicin-induced mice

To determine whether HydroMg modulates doxorubicin-induced apoptotic signaling *in vivo*, we performed TUNEL staining on the myocardial tissues. Compared with vehicle controls, doxorubicin significantly increased cardiomyocyte apoptosis, an effect substantially attenuated by HydroMg treatment (**Figure 5A** and **B**). Mechanistically, in acute models, doxorubicin elevated cardiac caspase-3 levels, whereas HydroMg treatment suppressed this activation (**Figure 5C** and **D**). Expression of Bcl-2, an anti-apoptotic regulator,^23^ decreased in doxorubicin-exposed myocardium. HydroMg administration reversed this suppression, restoring Bcl-2 toward control levels (**Figure 5C** and **E**). These findings indicate that HydroMg treatment confers cardioprotection by antagonizing doxorubicin-driven apoptotic cascades, thereby improving cardiac function.

**Figure 5:**
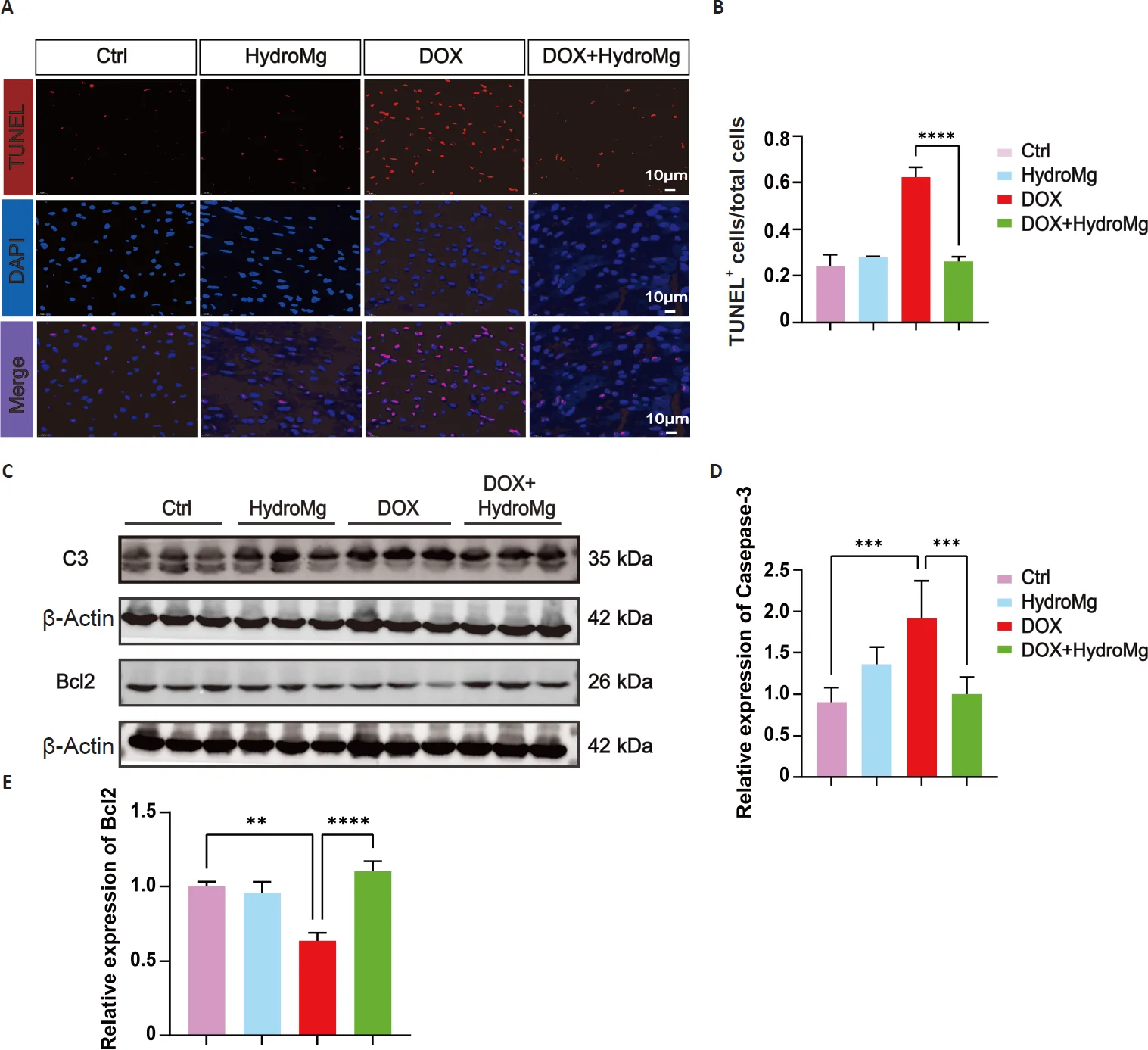
HydroMg attenuates cardiomyocyte apoptosis in mice with doxorubicin-induced myocardial injury. (A) TUNEL staining of cardiac tissue sections (*n* ≥ 3 per group). HydroMg attenuated doxorubicin-induced cardiomyocyte apoptosis. Scale bars: 10 μm. (B) Quantification of TUNEL-positive cardiomyocytes. (C) Western blot analysis of caspase-3 and Bcl2 protein expression levels in mice cardiac tissues. (D, E) Statistical analysis of relative protein expression levels for caspase-3 (D), and Bcl2 (E) (*n* = 3 per group). All results are expressed as mean ± SEM. Statistical significance was determined by one-way analysis of variance with Tukey’s *post hoc* test for multiple comparisons. ^**^P < 0.01, ^***^P < 0.001, ^****^P < 0.0001. Ctrl: Control; DAPI: 4’,6-diamidino-2-phenylindole; DOX: doxorubicin; TUNEL: terminal deoxynucleotidyl transferase dUTP nick-end labeling.

### HydroMg mitigates doxorubicin-induced mitochondrial dysfunction and attenuates ferroptosis in the heart of doxorubicin-induced mice

To evaluate the protective effects of HydroMg against doxorubicin-induced mitochondrial dysfunction *in vivo*, myocardial tissues were isolated from mouse hearts. Western blot analysis showed that HydroMg administration significantly upregulated the expression of TOM20 (**Figure 6A** and **B**), which is a mitochondrial outer membrane receptor, indicating enhanced mitochondrial function. To assess mitochondrial respiratory chain integrity, we quantified the protein levels of complexes I-V (CI-CV). HydroMg treatment significantly attenuated the doxorubicin-induced decline in complexes II (CII), III (CIII), and V (CV) in cardiac tissue (**Figure 6C** and **D**). Furthermore, to delineate the molecular mechanisms underlying HydroMg-mediated mitochondrial improvement, we analyzed ferroptosis regulators. Western blotting revealed that doxorubicin significantly downregulated GPX4 and SLC7A11 expression compared with controls, whereas HydroMg treatment robustly restored the levels of these key ferroptosis suppressors (**Figure 6E–H**). Thus, HydroMg therapy mitigates mitochondrial dysfunction to reduce ferroptosis in doxorubicin-induced cardiomyopathy, thereby exerting cardioprotective effects.

**Figure 6:**
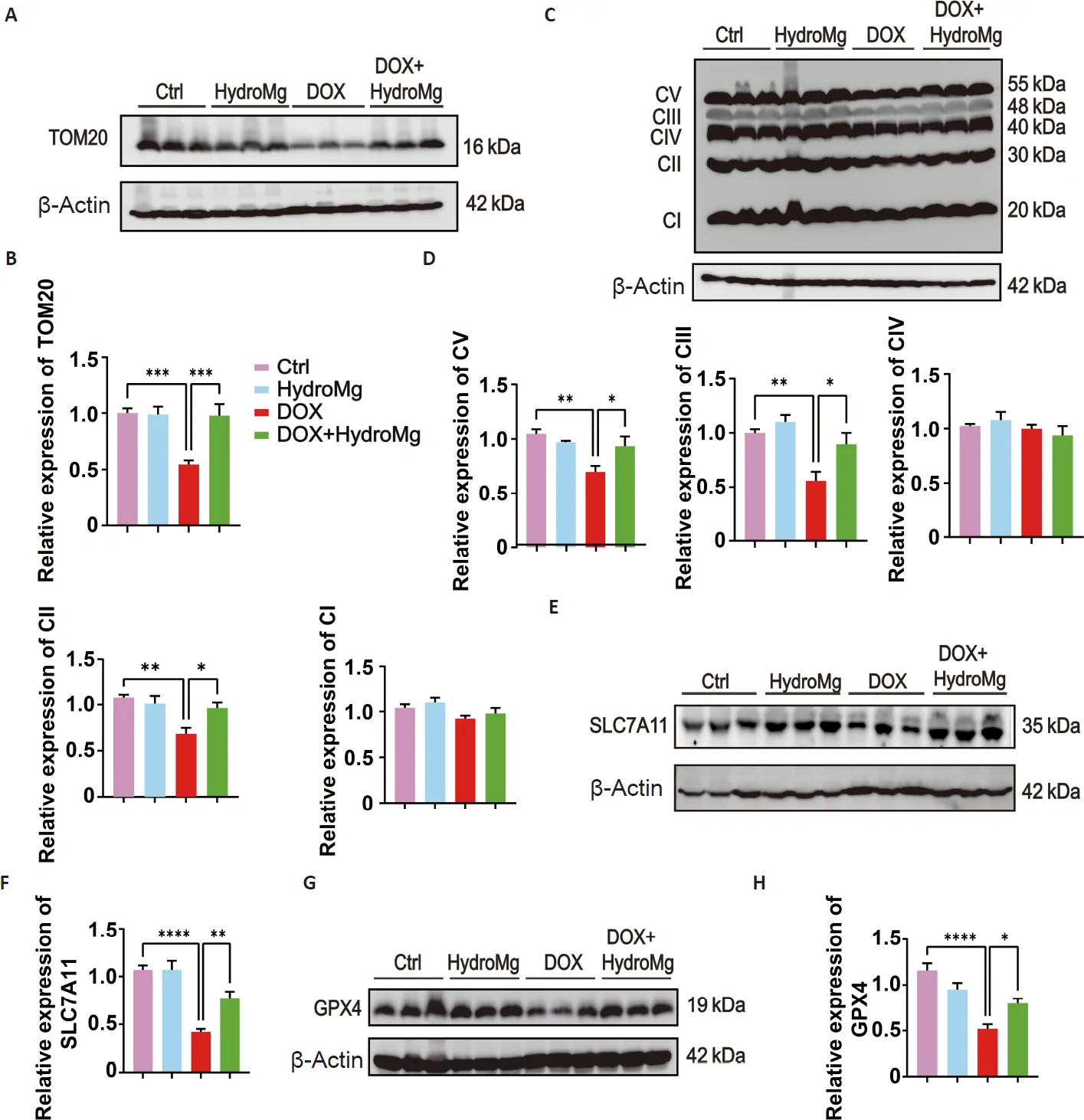
HydroMg mitigates doxorubicin-induced mitochondrial dysfunction and attenuates ferroptosis. (A, B) Western blot analysis of TOM20 protein expression levels in mouse cardiac tissues (A) and statistical analysis (B). (C, D) Western blot analysis of protein expression levels for CI, CII, CIII, CIV, and CV in mouse cardiac tissues (C) and statistical analysis (D). (E, F) Western blot analysis of SLC7A11 protein expression levels in mouse cardiac tissues (E) and statistical analysis (F). (G, H) Western blot analysis of GPX4 protein expression levels in mouse cardiac tissues (G) and statistical analysis (H). All results are presented as mean ± SEM (*n* = 3 per group). ^*^P < 0.05, ^**^P < 0.01, ^***^P < 0.001, ^****^P < 0.0001 (one-way analysis of variance followed by Tukey’s *post hoc* test). CI: Complex I; CII: Complex II; CIII: Complex III; CIV: Complex IV; Ctrl: control; CV: Complex V; DOX: doxorubicin; GPX4: glutathione peroxidase 4; SLC7A11: solute carrier family 7 member 11; TOM20: translocase of the outer mitochondrial membrane 20.

## Discussion

Mitochondrial dysfunction is recognized as a central pathological mechanism underlying doxorubicin-induced cardiotoxicity.^24^ Our study demonstrates that doxorubicin induces mitochondrial ultrastructural damage and bioenergetic dysfunction, which contribute to ROS overproduction and oxidative phosphorylation impairment. These interconnected pathological events subsequently concurrently activate both apoptosis and ferroptosis pathways, ultimately resulting in progressive cardiomyocyte death and contractile dysfunction. HydroMg confers comprehensive cardioprotection by disrupting these doxorubicin-induced pathological cascades through mitochondrial stabilization. In doxorubicin-treated H9C2 cardiomyocytes, HydroMg co-administration at a concentration of 1 μg/mL, which was selected based on preliminary screening for its optimal pro-proliferative effect and biological relevance within the 0.1–10 μg/mL range, attenuated doxorubicin-induced cardiomyocyte death by inhibiting apoptotic signaling, reducing ROS production, and restoring mitochondrial membrane potential. Higher concentrations (e.g., 100 μg/mL) were excluded to avoid potential off-target or toxic effects. In doxorubicin-treated mice, HydroMg co-treatment significantly improved survival outcomes, restored cardiac function, suppressed caspase-3-dependent apoptosis, and attenuated ferroptosis through upregulation of SLC7A11 expression and GPX4 activity. These findings demonstrate that mitochondrial-targeted therapy with HydroMg offers a mechanism-driven strategy to counteract chemotherapy-induced cardiotoxicity.

Our data validate its cardioprotective efficacy against doxorubicin injury, achieved via synchronized hydrogen release and blockade of the pathological cascade. Mechanistically, HydroMg facilitates concerted ROS neutralization and mitochondrial stabilization, effectively attenuating oxidative stress and apoptosis. This is particularly notable given that doxorubicin selectively accumulates in cardiomyocyte mitochondria, inducing electron transport chain dysfunction, oxidative phosphorylation collapse, and calcium overload-mediated mitochondrial permeability transition pore activation.^25^,^26^,^27^ These events drive reactive ROS overproduction and mitochondrial failure through inner membrane destabilization involving cardiolipin complexes,^28^,^29^ ultimately culminating in apoptosis-ferroptosis crosstalk^30^ with cytochrome C release and cardiomyocyte death.^31^ Current therapeutic strategies targeting this pathology primarily involve mitochondria-targeted antioxidants (e.g., mitochondria-targeted quinone, carvedilol, berberine, dexrazoxane),^9^,^32^,^33^,^34^,^35^,^36^,^37^,^38^ vitamins,^39^ and molecular hydrogen.^40^ However, conventional antioxidants exhibit limited mitochondrial membrane permeability despite their efficacy.^41^,^42^ Molecular hydrogen,^43^,^44^ while capable of mitochondrial penetration, suffers from compromised targeting efficacy due to insufficient systemic bioavailability with current delivery systems. In contrast, HydroMg’s molecular encapsulation engineering minimizes drug leakage through structural optimization.

We showed that HydroMg exerts dual-pathway inhibition by concurrently blocking apoptosis (via caspase-3 suppression and Bcl-2 upregulation) and ferroptosis (through SLC7A11/GPX4 axis stabilization), providing a more comprehensive therapeutic approach for cell death interception.

Mitochondrial-derived ROS critically drive doxorubicin cardiotoxicity by propagating iron-dependent ferroptosis through lipid peroxidation cascades.^45^,^46^ This process initiates with doxorubicin-induced accumulation of labile iron pools,^47^ which catalyze oxidative damage and deplete antioxidant reserves (SLC7A11/GPX4),^48^,^49^ establishing a self-amplifying redox imbalance. The resultant lipid peroxidation products further exacerbate ROS generation, creating a pathogenic loop that combines with mitochondrial ROS overproduction to induce cardiomyocyte ferroptosis—a distinct iron-mediated cell death pathway contributing to progressive cardiac dysfunction.^17^,^50^,^51^,^52^,^53^,^54^ In line with our findings, Tadokoro et al.^55^ reported concurrent apoptosis and ferroptosis in doxorubicin-induced cardiomyopathy. Crucially, combinatorial inhibition using zVAD (an apoptosis blocker) and Fer-1 (ferroptosis inhibitor) achieved profound attenuation of cardiomyocyte death, highlighting the therapeutic necessity of the multi-pathway interception. Current therapeutic approaches show the inherent limitations of mono-target strategies.

This study has several limitations: the efficacy of HydroMg remains unvalidated in chronic doxorubicin cardiomyopathy models, there is a lack of mechanistic verification using primary cardiomyocytes, ferroptosis pathway crosstalk has not been explored via co-administration of inhibitors, and the exclusive use of male mice, while minimizing confounding effects of the estrous cycle, restricts the generalizability of the findings to females. These limitations will be addressed in future studies to validate HydroMg’s efficacy in diverse experimental models and across disease stages.

Collectively, our findings demonstrate that HydroMg treatment protects against doxorubicin-induced cardiac injury by restoring mitochondrial homeostasis via ROS scavenging. This restoration leads to coordinated suppression of apoptosis and ferroptosis, consequently promoting cardiac functional recovery.

## Additional files:

***Additional Figure 1:*** *Restoration of myocardial ATP levels by HydroMg treatment in DOX-induced cardiac injury.*

Additional Figure 1 Restoration of myocardial ATP levels by HydroMg treatment in DOX-induced cardiac injury.
All results are expressed as mean ± SEM (*n* ≥ 3 per group). ****P < 0.0001 (one-way analysis of variance followed by Tukey's *post hoc* test). ATP: Adenosine triphosphate; Ctrl: control; DOX: doxorubicin.

***Additional Figure 2:*** *HydroMg preserves cardiac function against DOX-induced impairment.*

Additional Figure 2 HydroMg preserves cardiac function against DOX-induced impairment.
(A) Ejection fraction. (B) Fractional shortening. All results are expressed as mean ± SEM (*n* ≥ 3 per group). ***P < 0.001 (one-way analysis of variance followed by Tukey’s *post hoc* test). Ctrl: Control; DOX: doxorubicin; ns: not significant.

## Data Availability

*All data relevant to the study are included in the article or uploaded as additional files.*

## References

[1] Chang HY, Hsu HC, Fang YH, Liu PY, Liu YW (2024). Empagliflozin attenuates doxorubicin-induced cardiotoxicity by inhibiting the JNK signaling pathway. Biomed Pharmacother.

[2] Chatterjee K, Zhang J, Honbo N, Karliner JS (2010). Doxorubicin cardiomyopathy. Cardiology.

[3] Avagimyan A, Pogosova N, Kakturskiy L (2024). Doxorubicin-related cardiotoxicity: review of fundamental pathways of cardiovascular system injury. Cardiovasc Pathol.

[4] Sahil J, Shirish RS, Sangmi K, Susan J, David DC (2024). Hydroxychloroquine as a potential therapy for ANCA-associated vasculitis. Vessel Plus.

[5] Kelly P, David W, Michael M (2023). Efficacy and safety of a 0/1-h high-sensitivity cardiac troponin I (hs-cTnI) protocol to rule out acute coronary syndrome over a one-year period. Vessel Plus.

[6] Tai P, Chen X, Jia G (2023). WGX50 mitigates doxorubicin-induced cardiotoxicity through inhibition of mitochondrial ROS and ferroptosis. J Transl Med.

[7] Swain SM, Whaley FS, Ewer MS (2003). Congestive heart failure in patients treated with doxorubicin: a retrospective analysis of three trials. Cancer.

[8] Wu L, Wang L, Du Y, Zhang Y, Ren J (2023). Mitochondrial quality control mechanisms as therapeutic targets in doxorubicin-induced cardiotoxicity. Trends Pharmacol Sci.

[9] Wallace KB, Sardão VA, Oliveira PJ (2020). Mitochondrial determinants of doxorubicin-induced cardiomyopathy. Circ Res.

[10] Songbo M, Lang H, Xinyong C, Bin X, Ping Z, Liang S (2019). Oxidative stress injury in doxorubicin-induced cardiotoxicity. Toxicol Lett.

[11] Hu C, Zhang X, Wei W (2019). Matrine attenuates oxidative stress and cardiomyocyte apoptosis in doxorubicin-induced cardiotoxicity via maintaining AMPKα/UCP2 pathway. Acta Pharm Sin B.

[12] Chen S, Chen J, Du W (2023). PDE10A inactivation prevents doxorubicin-induced cardiotoxicity and tumor growth. Circ Res.

[13] Kalyanaraman B (2020). Teaching the basics of the mechanism of doxorubicin-induced cardiotoxicity: have we been barking up the wrong tree?. Redox Biol.

[14] Kong CY, Guo Z, Song P (2022). Underlying the mechanisms of doxorubicin-induced acute cardiotoxicity: oxidative stress and cell death. Int J Biol Sci.

[15] Sun X, Chen H, Gao R (2023). Mitochondrial transplantation ameliorates doxorubicin-induced cardiac dysfunction via activating glutamine metabolism. iScience.

[16] Vitale R, Marzocco S, Popolo A (2024). Role of oxidative stress and inflammation in doxorubicin-induced cardiotoxicity: a brief account. Int J Mol Sci.

[17] Christidi E, Brunham LR (2021). Regulated cell death pathways in doxorubicin-induced cardiotoxicity. Cell Death Dis.

[18] Yao L, Chen H, Wu Q, Xie K (2019). Hydrogen-rich saline alleviates inflammation and apoptosis in myocardial I/R injury via PINK-mediated autophagy. Int J Mol Med.

[19] Ying J, Zhang K, Huang Y (2025). Molecular hydrogen: mechanism against oxidative stress and application in periodontitis: a review. Medicine (Baltimore).

[20] Liu B, Xue J, Zhang M (2020). Hydrogen inhalation alleviates nonalcoholic fatty liver disease in metabolic syndrome rats. Mol Med Rep.

[21] Zhao M, Jin Z, Xia C (2023). Inhibition of free heme-catalyzed Fenton-like reaction prevents non-alcoholic fatty liver disease by hepatocyte-targeted hydrogen delivery. Biomaterials.

[22] National Research Council (2011). Guide for the Care and Use of Laboratory Animals: Eighth Edition.

[23] Czabotar PE, Garcia-Saez AJ (2023). Mechanisms of BCL-2 family proteins in mitochondrial apoptosis. Nat Rev Mol Cell Biol.

[24] Zeng X, Zhang H, Xu T (2024). Vericiguat attenuates doxorubicin-induced cardiotoxicity through the PRKG1/PINK1/STING axis. Transl Res.

[25] Ghigo A, Li M, Hirsch E (2016). New signal transduction paradigms in anthracycline-induced cardiotoxicity. Biochim Biophys Acta.

[26] Schlame M, Rua D, Greenberg ML (2000). The biosynthesis and functional role of cardiolipin. Prog Lipid Res.

[27] Takemura G, Fujiwara H (2007). Doxorubicin-induced cardiomyopathy from the cardiotoxic mechanisms to management. Prog Cardiovasc Dis.

[28] Ichikawa Y, Ghanefar M, Bayeva M (2014). Cardiotoxicity of doxorubicin is mediated through mitochondrial iron accumulation. J Clin Invest.

[29] Goormaghtigh E, Huart P, Praet M, Brasseur R, Ruysschaert JM (1990). Structure of the adriamycin-cardiolipin complex. Role in mitochondrial toxicity. Biophys Chem.

[30] Damiani RM, Moura DJ, Viau CM, Caceres RA, Henriques JAP, Saffi J (2016). Pathways of cardiac toxicity: comparison between chemotherapeutic drugs doxorubicin and mitoxantrone. Arch Toxicol.

[31] Zhang X, Hu C, Kong CY (2020). FNDC5 alleviates oxidative stress and cardiomyocyte apoptosis in doxorubicin-induced cardiotoxicity via activating AKT. Cell Death Differ.

[32] Zhao Y, Tian X, Liu G, Wang K, Xie Y, Qiu Y (2019). Berberine protects myocardial cells against anoxia-reoxygenation injury via p38 MAPK-mediated NF-κB signaling pathways. Exp Ther Med.

[33] Kametani R, Miura T, Harada N (2006). Carvedilol inhibits mitochondrial oxygen consumption and superoxide production during calcium overload in isolated heart mitochondria. Circ J.

[34] Oliveira PJ, Esteves T, Rolo AP, Palmeira CM, Moreno AJ (2004). Carvedilol inhibits the mitochondrial permeability transition by an antioxidant mechanism. Cardiovasc Toxicol.

[35] Deng S, Yan T, Jendrny C (2014). Dexrazoxane may prevent doxorubicin-induced DNA damage via depleting both topoisomerase II isoforms. BMC Cancer.

[36] Lue Y, Gao C, Swerdloff R (2018). Humanin analog enhances the protective effect of dexrazoxane against doxorubicin-induced cardiotoxicity. Am J Physiol Heart Circ Physiol.

[37] Hasinoff BB, Patel D, Wu X (2003). The oral iron chelator ICL670A (deferasirox) does not protect myocytes against doxorubicin. Free Radic Biol Med.

[38] Xiong C, Wu YZ, Zhang Y (2018). Protective effect of berberine on acute cardiomyopathy associated with doxorubicin treatment. Oncol Lett.

[39] Saengsin K, Sittiwangkul R, Chattipakorn SC, Chattipakorn N (2023). Hydrogen therapy as a potential therapeutic intervention in heart disease: from the past evidence to future application. Cell Mol Life Sci.

[40] Ohta S (2012). Molecular hydrogen is a novel antioxidant to efficiently reduce oxidative stress with potential for the improvement of mitochondrial diseases. Biochim Biophys Acta.

[41] Szeto HH (2006). Mitochondria-targeted peptide antioxidants: novel neuroprotective agents. AAPS J.

[42] Laura EV, Albert R, María Nazarena P, Juan José GL, Benito A, Nuria FH (2023). Complex pathogens in infective endocarditis. Vessel Plus.

[43] Johnsen HM, Hiorth M, Klaveness J (2023). Molecular hydrogen therapy-a review on clinical studies and outcomes. Molecules.

[44] Wu C, Zou P, Feng S (2023). Molecular hydrogen: an emerging therapeutic medical gas for brain disorders. Mol Neurobiol.

[45] Zheng J, Conrad M (2020). The metabolic underpinnings of ferroptosis. Cell Metab.

[46] Kitakata H, Endo J, Ikura H (2022). Therapeutic targets for DOX-induced cardiomyopathy: role of apoptosis vs. ferroptosis. Int J Mol Sci.

[47] Chen Y, Guo X, Zeng Y (2023). Oxidative stress induces mitochondrial iron overload and ferroptotic cell death. Sci Rep.

[48] Yi X, Wang Q, Zhang M, Shu Q, Zhu J (2024). Ferroptosis: a novel therapeutic target of natural products against doxorubicin-induced cardiotoxicity. Biomed Pharmacother.

[49] Zhang H, Pan J, Huang S (2024). Hydrogen sulfide protects cardiomyocytes from doxorubicin-induced ferroptosis through the SLC7A11/GSH/GPx4 pathway by Keap1 S-sulfhydration and Nrf2 activation. Redox Biol.

[50] Li J, Cao F, Yin HL (2020). Ferroptosis: past, present and future. Cell Death Dis.

[51] Liang D, Minikes AM, Jiang X (2022). Ferroptosis at the intersection of lipid metabolism and cellular signaling. Mol Cell.

[52] Li J, Jia YC, Ding YX, Bai J, Cao F, Li F (2023). The crosstalk between ferroptosis and mitochondrial dynamic regulatory networks. Int J Biol Sci.

[53] Dixon SJ, Lemberg KM, Lamprecht MR (2012). Ferroptosis: an iron-dependent form of nonapoptotic cell death. Cell.

[54] Friedmann Angeli JP, Schneider M, Proneth B (2014). Inactivation of the ferroptosis regulator Gpx4 triggers acute renal failure in mice. Nat Cell Biol.

[55] Tadokoro T, Ikeda M, Ide T (2020). Mitochondria-dependent ferroptosis plays a pivotal role in doxorubicin cardiotoxicity. JCI Insight.

